# Next-Generation Sequencing Whole-Genome Analysis for Targeted Treatment Approach of Metastatic Bartholin Gland Adenocarcinoma: An Emblematic Case Report and Review of the Literature

**DOI:** 10.3390/diagnostics11112085

**Published:** 2021-11-10

**Authors:** Antonio Macciò, Clelia Donisi, Elisabetta Sanna, Giacomo Chiappe, Sonia Nemolato, Luca Melis, Sara Oppi, Brunella Mola, Clelia Madeddu

**Affiliations:** 1 Department of Gynecologic Oncology, A. Businco Hospital, ARNAS G. Brotzu, 09100 Cagliari, Italy; dr.elisabettasanna@gmail.com (E.S.); giacomo.chiappe@aob.it (G.C.); 2 Department of Surgical Sciences, University of Cagliari, 09100 Cagliari, Italy; 3 Department of Medical Sciences and Public Health, Medical Oncology Unit, Azienda Ospedaliero Universitaria di Cagliari, University of Cagliari, 09042 Monserrato, Italy; cleliadonisi@gmail.com (C.D.); clelia.madeddu@unica.it (C.M.); 4 Department of Pathology, ARNAS G. Brotzu, 09100 Cagliari, Italy; sonia.nemolato@aob.it; 5 Department of Nuclear Medicine, A. Businco Hospital, ARNAS G. Brotzu, 09100 Cagliari, Italy; lucamelis@aob.it; 6 Hematology and Transplant Center, A. Businco Hospital, ARNAS G. Brotzu, 09100 Cagliari, Italy; sara.oppi@gmail.com (S.O.); clelia_md@yahoo.it (B.M.)

**Keywords:** vulvar cancer, Bartholin gland adenocarcinoma, minimally invasive surgery, whole genomic sequencing, immunotherapy, mTOR inhibitors, targeted therapy

## Abstract

Bartholin gland adenocarcinoma (BGA) is extremely rare and is characterized by high rates of lymph-node recurrence and distant metastases. No effective palliative treatments are available for metastatic BGA; therefore, advanced BGA remains a challenge for gynecologic oncologists. Considering the rarity of this disease and the lack of a standardized approach, the present study aims to discuss the available literature on current therapies for BGA and to describe an emblematic case treated with a novel tailored approach. A postmenopausal woman with advanced BGA was referred to our department for an adequate evaluation, staging and treatment. Notably, we used PET/CT as a fundamental imaging technique for staging and follow-up. The patient underwent primary surgery followed by standard chemotherapy and pelvic radiotherapy. Three months later, she relapsed, with the appearance of multiple metastatic sites. Considering the evident chemoresistance to standard chemotherapy and the absence of valid therapeutic alternatives for this rare cancer, she was treated with a combination of repeated minimally invasive surgical procedures for all the resectable metastatic lesions and innovative approaches comprising, firstly, chemoimmunotherapy with Nivolumab combined with metronomic vinorelbine, which resulted in a clinical response for approximately 7 months. Upon disease progression, we used a targeted systemic approach based on the whole genomic profile of the primary tumor, which showed *PTEN* loss, which is predictive of a benefit from an mTOR inhibitor, and a *CCND1* amplification, which predicts sensitivity to CDK4/6 inhibitors. Therefore, she received Everolimus, resulting in a significant metabolic response that lasted 12 months. Thereafter, upon further progression of the disease, the patient started Palbociclib treatment, which is currently ongoing, with evidence of a metabolic response. The patient has survived for 54 months from diagnosis, with a good performance status. In conclusion, the present paper confirms the lack of efficacy of conventional therapeutic regimens in the context of advanced, recurrent or metastatic adenocarcinomas of the Bartholin gland. The case report shows how a personalized multidisciplinary approach based on repeated minimally invasive surgery and tailored anticancer treatment based on whole-genome sequencing analysis could be effective and associated with prolonged survival in this rare gynecological cancer.

## 1. Introduction

Bartholin gland (BG) carcinoma is an extremely rare condition, accounting for <1% of female genital malignancies and <2% of vulvar cancers [1]. BG carcinoma usually occurs in postmenopausal women and occurs at a younger age than non-Bartholin-gland-related vulvar carcinoma [2,3].

A tumor is accepted as being a primary tumor of the BG when (1) the tumor involving the area of the BG is histologically compatible with an origin from this organ, (2) areas of apparent transition from normal to neoplastic elements are found in its pathology, and (3) there is no evidence of a primary tumor elsewhere. BGC is staged as vulvar cancer and is considered a median vulvar tumor [4].

Embryologically, the Bartholin glands (BG) originate from the urogenital sinus and are composed of several types of epithelium. The most common carcinomas reported are squamous cell carcinomas (SCCs), adenoido-cystic carcinoma (AAC) and adenocarcinoma, the last accounting for approximately 25% of cases [4]. Bartholin gland adenocarcinoma (BGA) originates from the mucin-secreting columnar epithelial cells of the acini and is identified by the absence of the uniform acinar pattern of adenoid cystic carcinoma (ACC) [5,6]. Primary adenocarcinomas of the vulva are very rare; the majority of these arise within the Bartholin gland.

BGAs are often characterized by an infiltrative pattern, and, in 30–55% of cases, the inguinofemoral lymph nodes show metastasis. Upon the diagnosis of BGA, all patients should undergo evaluation to determine the extent of the local and metastatic disease. The global literature to date indicates that there are no satisfactory imaging modalities for evaluating the extent of the disease and possible deep pelvic lymph node metastasis [5]. The standard of care for this rare tumor is surgery—radical local excision plus inguinofemoral lymphadenectomy [1]. Additionally, adjuvant radiotherapy is recommended when the lymph nodes are involved or positive surgical margins are indicated in the pathology. The inherently aggressive nature of this malignancy warrants systemic adjuvant therapy; however, due to the limited available data, which are only derived from case reports or retrospective studies, which are highly variable in their treatment approaches, determining the best treatment regimen remains difficult. Therefore, there is no consolidated evidence to guide clinical practice, leading to subjective decisions regarding treatment [2,5].

The rate of relapse is high and varies depending on the histotype [5]. At its initial stage, BG carcinoma has a good rate of curability; however, metastatic BG carcinoma has a very poor prognosis, with a 5-year survival rate lower than 20%; the rate of survival is even lower in the case of the BGA histotype, mainly due to the lack of effective therapeutic options [5]. Indeed, the literature is largely focused on the SCC and AAC histotypes [2,7,8], and only a few case reports and small retrospective studies propose a specific chemotherapy regimen for BGA [9,10]. BGA is characterized by a high rate of distant metastases that are observed especially in women with inguinal node metastasis at diagnosis, and a high rate of lymph-node recurrence [2,10]. There are currently no standardized treatment approaches for advanced and recurrent BGA, due to a lack of available data to inform specific therapeutic guidelines. Some data suggest no benefit from chemotherapy and contraindications for additional radiotherapy in the case of recurrence [5]. Moreover, no studies on valid chemotherapy as a a palliative treatment are available for patients with metastatic BGA [2,10]. Therefore, treatment options should be considered on a case-by-case basis, and BGA subsequently remains a challenge for gynecologic oncologists. Precision medicine such as approaches utilizing comprehensive genome sequencing can provide useful information for establishing a tailored biological approach to treating this uncommon and difficult-to-treat disease.

The aim of the present study was to present the literature available on the management of metastatic BGA and, considering the rarity of this disease, to report an emblematic case of a woman with metastatic BGA who relapsed following primary surgery and chemoradiotherapy and who was treated with a combination of repeated minimally invasive surgical procedures and a targeted systemic approach based on her genomic profile, with an impressive overall survival of 54 months.

## 2. Case Report

**Figure 1:**
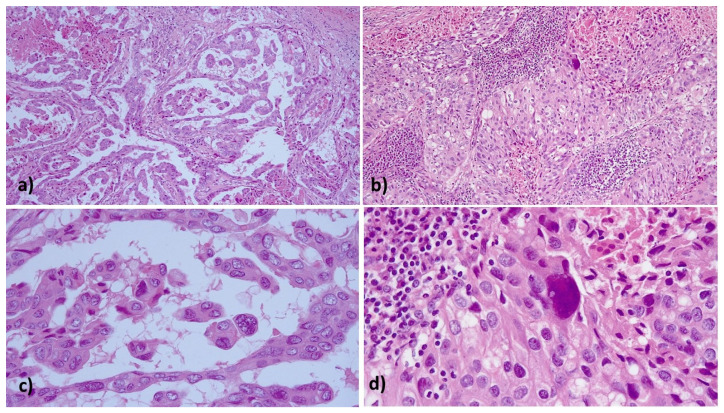
Histopathological findings: (**a**) papillary architecture of Bartholin gland carcinoma with necrosis (HE 200×); (**b**) infiltrating nest of poorly differentiated adenocarcinoma with solid and glandular pattern (HE 200×); (**c**,**d**) moderate to severe nuclear atypia with eosinophilic cytoplasm in the papillary and solid architecture (HE 400×).

A 63-year-old Caucasian woman, para 1, with an unremarkable medical, surgical and familiar history, was admitted to our Department of Gynecologic Oncology, Businco Hospital, coming from another institute, where, for the appearance of an asymptomatic inguinal lump of 15 mm, on June 2017, she underwent a left inguinal lymph node biopsy. The pathology was consistent with metastasis of adenocarcinoma, and immunohistochemistry showed a Ki67 >50%, positive stain for cytokeratin 7 and negative stains for p16, cytokeratin 20, estrogen/progesterone receptor, CA125 and TTF1. The previous treating physician proposed a chemotherapy regimen to the patient and did not proceed with further diagnostic and staging procedures. The patient then chose to attend our department for further examination and possible treatment as appropriate. A medical examination was performed, which revealed the presence of inguinal metastatic disease and of an irregularly fixed, hard lesion in the BG area; a PET/CT showed high metabolic activity in the left BG, the left inguinal region and the left obturator lymph-nodes. On 12 September 2017, we performed the surgical resection of the tumor including the left labia majora, the left superficial and deep inguinal lymphadenectomy, and, using a laparoscopic approach, a left iliac lymphadenectomy and a left obturator lymphadenectomy including the excision of a bulky left obturator lymph-nodal mass (measuring approximately 3 cm). Pathology (Figure 1) showed the presence of papillary BGA of grade 3, muscle focally infiltrating with tumor-free resection margins, massive metastases of inguinal lymph-nodes with necrosis overcoming the capsule, metastasis in one out of 11 metastatic lymph nodes in the obturator fossa, and left iliac lymph nodes free from metastasis (0/12) (Stage IV).

**Figure 2:**
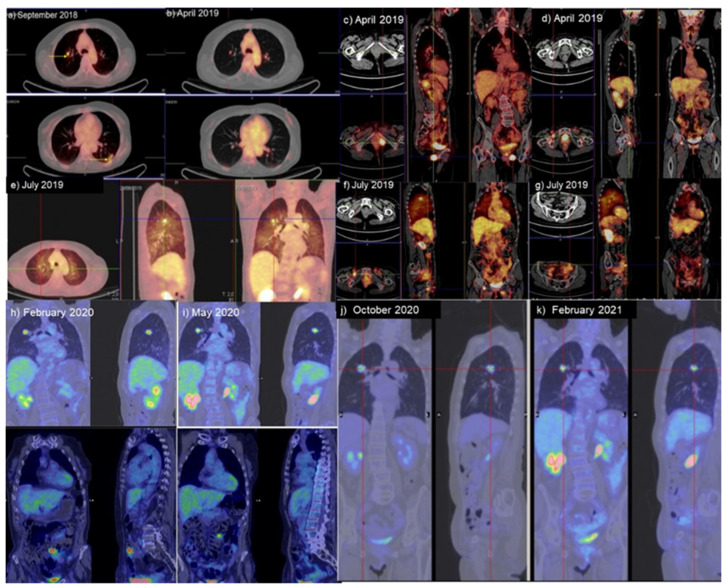
PET/CT during disease course: (**a**) In September 2018, PET/CT showed the appearance of hypermetabolic bilateral lung nodules; (**b**) the PET/CT scans of April 2019 after 6 months of Nivolumab showed a significant reduction in and disappearance of hypermetabolic activity of the lung lesions; (**c**,**d**) PET/CT scan on April 2019 showed stable hypermetabolic activity in the left perineum and vagina and hypermetabolic activity in the right inguinal region; (**e**–**g**) in July 2019, imaging showed progression of disease in the lung (**e**) in comparison to April 2019, as well as the appearance of hypermetabolic areas in the left anterior abdominal wall (**f**) and in the right inguinal region (**g**); (**h**,**i**) PET/CT imaging on February (**h**) and May 2020 (**i**) after 6 and 9 months of treatment with mTOR inhibitor, respectively; (**j**,**k**) PET/CT imaging in October 2021 and February 2021 before and after 3 months of treatment with Palbociclib.

The patient was then started on a weekly doublet chemotherapy regimen: cisplatin at 50 mg/m^2^ with Taxol at 90 mg/m^2^. Then, she underwent a PET scan for follow-up at 3 months (November 2017): high metabolic activity was observed in the right common iliac (near the aortoiliac carrefour) and in the obturator fossa. She subsequently underwent surgery: the bulky obturator and pericaval iliac lymph nodes were observed, and, after an extemporaneous histological examination that confirmed the metastatic nature, the exeresis of the iliac, lombo-aortic and obturator bulky nodal masses was performed. Considering the clear chemoresistance to the standard chemotherapy regimen utilized before, a closed periodical follow-up with PET/CT was planned. At the next follow-up on February 2018, the PET/CT showed high metabolic activity in the pelvis between the left emisacral region and the left iliac vessels. Thus, the patient received radiotherapy from March to May 2018: the pelvis was irradiated with 60 Gy in 35 fractions; the radiotherapy resulted in severe neutropenia (G3). A PET/CT on June 2018 showed a reduction in the metabolic activity in the pelvis. In the next follow-up on September 2018, the PET/CT scan confirmed a reduction in metabolic activity in the pelvis but showed the appearance of hypermetabolic irregular areas between the left external obturator muscle and the left pectineus muscle and into the internal oblique muscle of the right anterior abdominal wall, as well as the appearance of hypermetabolic lung nodules in the superior and inferior lobes of both lungs (Figure 2a). At the gynecological clinical visit, the presence of a nodular lesion was observed in the vaginal and perineal regions near the site of the previous surgery. The patient thereafter underwent surgical excision of the symptomatic nodules in the vagina and in the left perineum, as well as of the lesion located in the right abdominal wall; the histological examination confirmed the presence of metastasis of adenocarcinoma.

In the absence of valid therapeutic alternatives and referring to some data in the literature indicating a benefit with anti-PD1 inhibitors in vulvar cancer [11], we opted for a combined chemoimmunotherapy approach with oral metronomic Navelbine plus Nivolumab at 240 mg, every 14 days, on 15 September 2018. After 14 cycles of chemoimmunotherapy on February 2019, the PET/CT scan showed the disappearance of the previously described hypermetabolic area in the right abdominal wall site of the surgical excision, a reduction in the hypermetabolic activity in the lung and the persistence of hypermetabolic findings in the perineum. These findings were indicative of a partial response to treatment; the patient therefore continued the same regimen. A further monitoring PET was conducted in April 2019, which showed a further significant reduction in metabolic activity in the superior lobe of the right lung, which was considered indicative of a response to treatment (Figure 2b–d). The patient then continued the chemoimmunotherapy until July 2019, when a PET/CT showed an increase in the hypermetabolic activity in the superior lobe of the right lung, in the left inferior lobe of the left lung, and in the perineum and vagina, as well as the appearance of hypermetabolic areas in the left anterior abdominal wall and in the right inguinal region. A detailed ultrasound evaluation of the areas that were indicated by the PET in the left abdominal wall identified two hyperecogenic lesions with irregular/gradient margins, each one measuring approximately 0.5 cm (Figure 2e–g).

**Figure 3:**
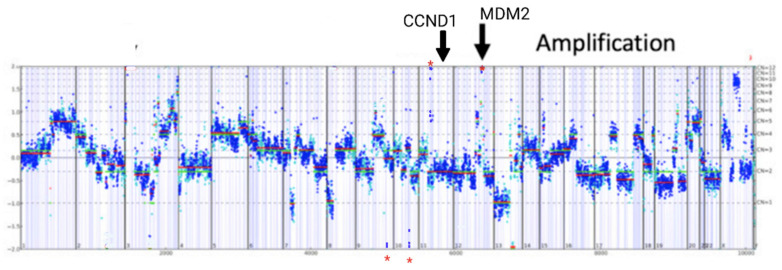
Comprehensive genomic profiling revealed multiple potentially targetable gene amplifications in *CCND1*, *FGF4* and *MDM2*. Y-axes denote log-ratio measurements of coverage obtained in test samples versus a normal reference sample, with assessed copy numbers marked by dashed lines. Each point denotes a genomic region measured by means of the assay (blue, exon; cyan, SNP), and these are ordered by genomic position. Red lines indicate the average log-ratio in a segment, whereas green lines illustrate the model prediction. Asterisks denote the detected *CCND1* (chr11) and *MDM2* amplification (chr12).

The disease was considered to have progressed, and in the absence of further valid options for treatment, the patient underwent comprehensive genomic profiling through a next-generation-sequencing (NGS)-based assay (FoundationOne^®^CDx) to identify potentially actionable targets [12]. The primary tumor sample was submitted to FoundationOne for sequencing and analysis: DNA was extracted from a formalin-fixed, paraffin-embedded tumor sample using a single DNA extraction method. Then, 50–1000 ng of tumor specimen was used for whole-genome shotgun library construction and the hybridization-based capture of all the coding exons from 309 cancer-related genes, one promoter region, one non-coding RNA and selected intronic regions from 34 commonly rearranged genes, 21 of which also include the coding exons. The assay therefore included the detection of alterations in a total of 324 genes. Using an Illumina^®^ HiSeq platform, libraries selected via hybrid capture were sequenced to a high uniform depth (targeting >500 median coverage with >99% of exons having a coverage >100×). The methods are described in detail in Appendix A. The gene alterations and variants of unknown significance that were found are listed in Table 1 and Table 2; an original data report is contained in Appendix B. Among them are gene mutations that represent potential actionable targets (Figure 3).

In detail, the loss of *PTEN*’s exons 2–5 may predict sensitivity to mTOR inhibitors [13], and *CCND1* amplification may predict sensitivity to CDK4/6 inhibitors [14]. The analysis also revealed an *MDM2* amplification, a microsatellite status-stable profile and a low mutational burden (3 muts/Mb); these results have been described to be associated with a low rate of clinical benefit from immunotherapy with immune-checkpoint inhibitors [15].

**Figure 4:**
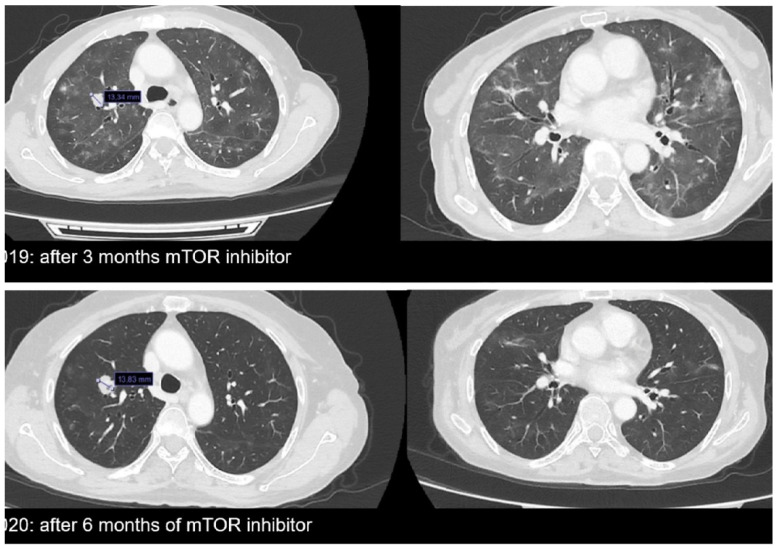
Computed tomography imaging in October 2019 and January 2020 during mTOR-inhibitor treatment.

Hence, the patient started taking Everolimus at 10 mg daily on August 2019; the dosage was reduced to 5 mg daily on October 2019, owing to pulmonary toxicity associated with cough and dyspnea and confirmed by CT-scan findings (Figure 4) of a nonhomogeneous parenchymal thickening compatible with mTOR-inhibitor-associated pneumonitis [16]. After 3 months of treatment, the PET/CT scan on 8 November 2019 confirmed persistent, metabolically stable disease activity in the right lung and a reduction in the metabolic activity in the right inguinal region, vagina and perineum. The patient continued taking Everolimus at 5 mg daily. A monitoring CT scan was carried out on 30 January 2020 (Figure 4). The results showed a reduction in the mass in the superior lobe of the right lung with two small cavitations, as well as an improvement in the indirect sign of pulmonary toxicity.

**Figure 5:**
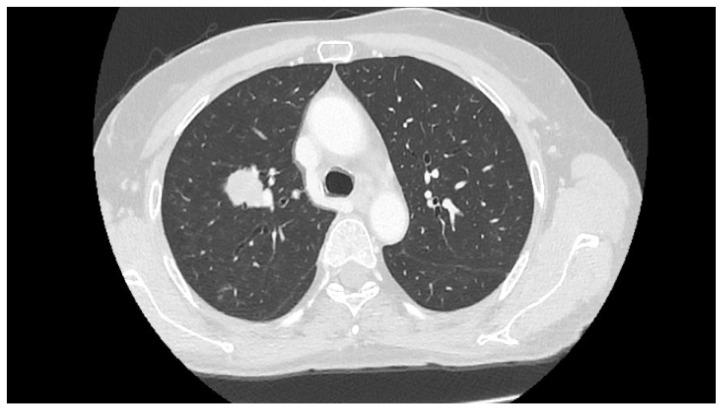
CT imaging on September 2020. Progression of disease with increase in size of the lung mass.

Then, a PET/CT scan performed on 18 February 2020 confirmed a reduction in the hypermetabolic areas in the superior lobe of the right lung and in the left abdominal wall, and a further reduction was observed in the vagina and perineum, with unchanged metabolic findings in the right inguinal region (Figure 2h). A subsequent PET scan carried out in May 2020 showed a further significant reduction in the hypermetabolic activity in the lung and disappearance of the hyperactivity in the left abdominal wall, with the remaining findings unchanged (Figure 2i). The patient then continued treatment with the mTOR inhibitor. After 12 months of treatment, the CT scan performed for disease monitoring on 24 September 2020 showed an increase in lung mass (approximately 28%) (Figure 5), whereas the remaining findings were unchanged. A PET/CT scan was performed in October 2020 that confirmed a significant increase in hypermetabolic activity in the known metastasis of the superior lobe of the right lung, as well as in the right inguinal region (Figure 2j). Additionally, the persistence of reduced metabolic activity was found in the perineal region. On 17 November 2020, Everolimus was therefore discontinued, and a superficial and deep right inguinal lymphadenectomy was performed. Pathology confirmed metastasis from BGA. Subsequently, always considering the genomic profile, the patient was started on a new targeted therapy with a CDK4/6 inhibitor (Palbociclib). A monitoring PET after 3 months of therapy in February 2021 showed a reduction in the size and number of hypermetabolic lung metastatic lesions (Figure 2k). The patient is currently undergoing treatment and is in good condition, without symptoms, with Performance Status 1 and Karnofsky 90; she is awaiting instrumental disease re-evaluation.

## 3. Discussion

Here, we report a case of metastatic primary BGA treated with an innovative approach, comprising a combination of multiple mini-invasive surgical procedures with immunotherapy, and thereafter a tailored systemic treatment based on the whole-genome sequencing profile of the patient. To our knowledge, this is the first case of BGA described in the literature for which detailed whole-genome sequencing analysis has been performed and for which consistent PET/CT has been used as the key imaging technique for staging and follow-up.

According to the literature, surgery represents the mainstay for resectable lesions in metastatic BG carcinoma [1,2]. Indeed, our patient underwent multiple surgical resections of each new resectable metastatic lesion through minimally invasive surgery, which enabled the disease to be controlled and the symptoms to be managed. These surgical resections could have significantly contributed to the prolonged overall survival of our patient.

By contrast, systemic chemotherapy is disappointing and is associated with poor outcomes [2,5]. Moreover, there are no data on effective antineoplastic drugs specific for BGA, which is even rarer and less studied than SCC [10,17]. The majority of studies in unselected cohorts of patients with vulvar cancers report the use of combined chemotherapy with cisplatin plus 5FU, which is likely to be more effective for SCC than for adenocarcinoma [5,10]. In particular, Cardosi et al. [10] reported a 15-year experience of 12 patients with primary BG carcinoma; 58.3% had Stage III/IV disease, and the majority received adjuvant radiation and/or chemotherapy. Six patients experienced recurrent disease, which was treated with heterogenous modalities alone or in combination, including surgery, radiation and, in only one case, chemotherapy with cisplatin plus 5FU [10]. Copeland et al. [17] reported disease recurrence in nine out of 36 patients with BG carcinoma, including six cases of BGA, over a 30-year period. Recurrent cases were treated with cyclophosphamide, radiotherapy or a combination of excision and RT or radical surgery and radiotherapy; none of the patients with positive nodes in whom disease recurred survived; two patients developed distant metastases and survived for 15 and 37 months, respectively.

Our case confirmed the lack of efficacy of conventional chemotherapy, which is typically indicated for the adenocarcinoma histotype. The development of a novel therapeutic regimen is warranted, and in this regard, the identification of potential effective immunotherapeutic approaches and targeted therapies could be critical for this rare disease [18].

Among potential innovative treatments, immunotherapy with anti-PD1 inhibitors (nivolumab and pembrolizumab) has been tested in vulvar carcinoma [19]. Nivolumab showed promising results, although mainly in squamous cell carcinoma and HPV-related cancer [11]. In a phase 1b basket trial, Pembrolizumab administered to patients with PDL-1-positive squamous vulvar cancer showed disappointing results, with an overall response rate of 6% and a median progression-free survival of 3.1 months [20]. In our case, we combined immunotherapy with metronomic chemotherapy, as reported in our previous studies [21,22]: in the present case, this approach led to disease control with a metabolic response through PET for approximately 7 months.

Notably, in our case study, a genomic NGS analysis was performed after the failure of the previous line of treatments, to define the mutational profile of the tumor and subsequently identify potential target-based therapeutic agents. The genomic analysis showed the following main actionable alterations: *PTEN* loss and *CCND1* amplification. The analysis also revealed an *MDM2* amplification, as well as a microsatellite status-stable profile and a low mutational burden (3 muts/Mb).

In the literature, there is a lack of data on specific gene mutations in BGA; the few data available come from analyses of vulvar cancer. In particular, Holway et al. [23] reported a high rate of *PTEN* mutation in vulvar carcinoma. Additionally, the mutation of *CCDN1* had previously been reported in some cases of vulvar cancer [24]. Other key genes frequently mutated in vulvar cancer, although not specifically in BGA, include *TP53*, *CDKN2A*, *BRCA1/2*, *PIK3CA*, *AKT1*, *HRAS*, *BRAF* and *HER2* [18]. A recent genomic-based study on vulvar cancer based on comprehensive genomic profiling described a 13.6% frequency of PTEN mutations in vulvar squamous cell carcinoma, but these mutations were much more frequent in HPV-positive cases [25]. This study showed a sharply different mutational profile between HPV-positive and HPV-negative vulvar cancers, highlighting how these are two different diseases and supporting the assessment of tandem HPV status and comprehensive genomic profiling to provide potentially effective therapies.

It has been also reported that some cases of BGA were mammary-like adenocarcinomas of the vulva, whereas true adenocarcinomas are rare [26]. However, no validated tests exist with which to distinguish between these [27]. Such tumors, including primary breast cancer, may show estrogen receptor and progesterone receptor expression or HER2 expression, or may be basal-like (triple negative) [28]. Presently, the discrimination between the basal-like type and the “true” adenocarcinoma is based only on the spot of the cancer cells in the vulva and the positivity of adenocarcinoma cells for CAM 5.2, Ca19-9, EMA, S-100, colporin, CD3, CD20, CD45RO and CEA [29]. In our case study, the evaluation of the immunohistochemistry profile could not allow the exclusion of a mammary-like adenocarcinoma; this could, at least in part, explain the presence (as in other BGA cases reported in the literature) of some somatic mutations found in breast cancer, such as *PTEN* loss.

The genomic alterations detected in our case study have been associated with the activity of certain targeted therapies approved for other tumor types. In particular, *PTEN* loss leads to the activation of the PI3K–AKT–mTOR pathway and can lead to uncontrolled cell growth and the suppression of apoptosis [30]. It may predict sensitivity to mTOR inhibitors [31], drugs that are already in use in other cancers (e.g., breast cancer). mTOR inhibitors have also been demonstrated to have cytotoxic effects on vulvar carcinoma cells in vitro [32]. Likewise, a target therapy is at hand for patients with amplifications in the *CCND1* gene, including CDK4/6 inhibitors [14], such as Abemaciclib, Ribociclib and Palbociclib, which we utilized in the present case. Notably, our patient had a long progression-free survival period with Everolimus, lasting approximately 12 months, even longer than that observed in the registrative trials of the drug in other diseases where the use is consolidated, such as metastatic hormone-positive, HER2-negative breast cancer [33,34]. Another mutation found in our case study, the loss of *CDKN2A* (and associated loss of p16INK4a function), has been correlated with sensitivity to CDK4/6 inhibitors in preclinical studies, as well as some case studies [35,36,37,38], although other clinical studies have shown no significant correlation [39,40]. However, to date, preliminary data on the outcomes of patients with metastatic vulvar cancer following a targeted therapy regimen are still scarce and disappointing. Fu et al. [41], in a limited population of 16 patients with metastatic or recurrent vulvar cancer treated with different targeted agents within a phase I trial program, reported a median overall survival of 4.4 months (range, 2.6–6.2 months). Notably, this series did not report any case with adenocarcinoma histology.

Overall, our patient showed a benefit from the targeted therapies used, particularly from the mTOR inhibitor, which resulted in a prolonged partial objective and metabolic response. Therefore, genomic tests that investigate the genetic mutations of a patient with BGA could be essential for tailoring the therapeutic approach, and further clinical data on the effectiveness of such targeted therapies in this very rare disease should be collected.

## 4. Conclusions

In conclusion, in the case study presented here, the choice of a treatment strategy comprising repeated surgical procedures for resectable disease, and targeted therapy based on the mutational profile of the patient were associated with prolonged survival and a clinical benefit to the patient. Notably, in our case, the role of surgery in controlling the disease and any associated symptoms cannot be ignored. Overall, this case study suggest how whole genomic profile could provide the basis for the development of a personalized therapy for managing a rare cancer, which, to date, remains bereft of a consensus on the most effective treatment approach. This remains a significant challenge, requiring novel approaches to be tested in further large prospective trials.

## Figures and Tables

**Table 1:** The genomic alterations detected in the patient’s tumor sample.

Genomic Signature	Result
Microsatellite status	MS-stable
Tumor mutational burden	3 mutations/Mb
**Gene (encoded protein)**	**Alteration**
*CCND1* (cyclin D1)	Amplification
*PTEN* (phosphatase and tensin homologue)	Loss of exons 2–5
*MDM2* (E3 ubiquitin protein ligase Mdm2)	Amplification
*CDKN2A/B* (p16INK4a, p14ARF, p15INK4b)	Loss
*FGF19* (fibroblast growth factor 19)	Amplification
*FGF3* (fibroblast growth factor 3)	Amplification
*FGF4* (fibroblast growth factor 4)	Amplification
*IKBKE* (I-kappa-B kinase epsilon)	Amplification
*MCL1* (myeloid cell leukemia 1)	Amplification
*MTAP* (S-methyl-5′-thioadenosine phosphorylase)	Loss
*NFKBIA* (NFkappaB inhibitor IkBA)	Amplification

**Abbreviations:** Mb, megabase; *MDM2*, murine double minute 2; *CDKN2A/B*, cyclin-dependent kinase inhibitor 2A/B.

**Table 2:** Variants of unknown significance (VUS) detected in the patient’s tumor sample.

Gene	Mutation
*BCL2*	E29Q
*CARD11*	R555W
*DNMT3A*	V501I
*ERBB3*	L1177I
*FAM123B*	H134P
*FANCA*	Rearrangement
*FGFR3*	V117I
*GNAS*	T415_G423del
*KDR*	V159M
*KIT*	E366D and rearrangement
*MED12*	Q2119_Q2120insHQQQ
*MSH3*	F71I
*PIK3C2B*	L889F
*SOCS1*	G122R

**Abbreviations:***BCL2*, B-cell lymphoma 2; *CARD11*, caspase recruitment domain-containing protein 11; *DNMT3A*, DNA methyltransferase-3a; *ERBB3*, erb-b2 receptor tyrosine kinase 3; *FANCA*, Fanconi anemia, complementation group A; *FGFR3*, fibroblast growth factor receptor 3; *KDR*, kinase insert domain receptor; *MED12*, mediator complex subunit 12; *MSH3*, MutS homolog 3; *PIK3C2B*, phosphatidylinositol-4-phosphate 3-kinase C2 domain; *SOCS1*, suppressor of cytokine signaling 1.

## Data Availability

Original clinical, laboratory and instrumental data can be found in the patient chart archived at the Department of Obstetrics and Gynecology and are available on request from the corresponding author.

## Appendix A

Test principles for genomic analysis

FoundationOne CDx was performed using DNA extracted from formalin-fixed, paraffin-embedded (FFPE) tumor samples. The proposed assay employed a single method for DNA extraction from an FFPE surgical resection specimen; 50–1000 ng of the DNA was used for whole-genome shotgun library construction and the hybridization-based capture of all the coding exons from 309 cancer-related genes, one promoter region, one non-coding RNA (ncRNA) and select intronic regions from 34 commonly rearranged genes, 21 of which also included the coding exons. The assay therefore included the detection of alterations in a total of 324 genes. Using an Illumina^®^ HiSeq platform, the hybrid capture-selected libraries were sequenced to a high uniform depth (targeting >500× median coverage with >99% of exons at a coverage >100×). The sequencing data were processed using a customized analysis pipeline designed to accurately detect all classes of genomic alterations, including base substitutions, indels, focal copy number amplifications, homozygous gene deletions and selected genomic rearrangements (e.g., gene fusions). Additionally, genomic signatures including loss of heterozygosity (LOH), microsatellite instability (MSI) and tumor mutational burden (TMB) were reported.

## Appendix B

Original data report from the next-generation sequencing-based assay (FoundationOne^®^CDx).
